# The impact of residential change and housing stability on recidivism: pilot results from the Maryland Opportunities through Vouchers Experiment (MOVE)

**DOI:** 10.1007/s11292-017-9317-z

**Published:** 2017-12-15

**Authors:** David S. Kirk, Geoffrey C. Barnes, Jordan M. Hyatt, Brook W. Kearley

**Affiliations:** 1 Department of Sociology & Nuffield College, University of Oxford, 1 New Road, Oxford, OX1 1NF UK; 2 Western Australia Police, Perth, Australia; 3 University of Cambridge, Cambridge, UK; 4 Drexel University, Philadelphia, PA USA; 5 University of Maryland, Baltimore, MD USA

**Keywords:** Housing, Residential change, Recidivism, Rearrest, Imprisonment, Harm reduction, Neighborhoods, Experiment, Randomized controlled trial, Collateral consequences of incarceration

## Abstract

**Objectives:**

This article provides a description and preliminary assessment of the Maryland Opportunities through Vouchers Experiment (MOVE), a randomized housing mobility program for former prisoners designed to test whether residential relocation far away from former neighborhoods, incentivized through the provision of a housing subsidy, can yield reductions in recidivism.

**Methods:**

The MOVE program was implemented as a randomized controlled trial. Participants were recruited from four different Maryland prisons and randomly assigned to experimental groups. In the first iteration of the experiment, treatment group participants received 6 months of free housing away from their home jurisdiction and control group participants received free housing back in their home jurisdiction. In the second iteration of the experiment, the treatment group remained the same and the control condition was redesigned to represent the status quo and did not receive free housing. Analyses were conducted of one-year rearrest rates.

**Results:**

With respect to reductions in recidivism, pilot results suggest that there is some benefit to moving and a benefit to receiving free housing. Rearrest was lower among the treatment group of movers than the non-movers, and was also lower for non-movers who received free housing versus non-movers who did not receive housing.

**Conclusions:**

To the extent that pilot results can be validated and replicated in a full-scale implementation of the MOVE program, policies that provide greater access to housing assistance for formerly incarcerated individuals may yield substantial public safety benefits, particularly housing opportunities located far away from former neighborhoods.

## Introduction

Roughly 1 in every 100 adults in the United States is in prison or jail at this very moment, with more than 1.5 million individuals serving time in state and federal prisons, and another 730,000 in local jails (Kaebel and Glaze 2016). An underappreciated fact of this era of mass incarceration is that most prisoners, 95% in fact, are eventually released from incarceration and returned to society (Langan and Levin 2002). More than 600,000 prisoners are released from U.S. prisons each year, and estimates suggest that two-thirds of these individuals will be rearrested and half will be reincarcerated within 3 years (Durose et al. 2014).

The public health consequences of high levels of criminal recidivism are dire. Recidivists tend to be high-rate criminal offenders who contribute substantially to the total volume of crime in a community (Hipp and Yates 2009). For instance, estimates reveal that active parolees account for between 15% and 28% of all arrests for violent crimes in the United States (Rosenfeld et al. 2005). Indeed, a relatively small proportion of active offenders account for the majority of crimes committed (Piquero et al. 2010; Sherman et al. 2016; Wolfgang et al. 1972). In turn, neighborhood crime, particularly violence, is a stressful condition that can have a variety of detrimental physical and mental health consequences for residents, including anxiety, depression, post-traumatic stress disorder, and impaired cognitive functioning (Aneshensel and Sucoff 1996; Buka et al. 2001; Margolin and Gordis 2000).

The fact that many released prisoners return home to the same environment with the same criminal opportunities and criminal peers that proved so detrimental to their behavior prior to incarceration is a likely contributor to high levels of recidivism. Indeed, recent estimates from Michigan suggest that the first post-prison place of residence for roughly one-third of newly released prisoners is within half a mile of their pre-prison place of residence, and that a full 60% reside within 5 miles of their pre-prison place of residence (Harding et al. 2013). Laub and Sampson’s (2003; Sampson and Laub 1993) life-course theory of desistance from crime provides a theoretical basis for understanding why returning to home neighborhoods is so detrimental and for developing interventions that can break the cycle of recidivism. Among the notable findings from their multi-decade project are that offenders desist from crime in response to structurally induced turning points, such as work and marriage. Turning points serve as catalysts for sustained behavioral change by providing opportunities for individuals to separate from the settings, situations, and criminal peers that facilitated their prior criminal behavior.

If criminal behavior is inextricably tied to social context, then residential change may be a turning point in the life course of crime, produced by separating individuals from those residential contexts associated with their previous criminality. Yet the mere act of moving is not likely as important as the magnitude of the move: the more separation an individual has from his or her criminal past—both geographically and socially—the better. Thus, moving next door may have little effect on behavioral change, but moving to an entirely different city may allow an individual to truly separate from her or his past and, therefore, lead to long-term behavioral change.^1^

To test whether voluntary residential relocation to a new city would lower the likelihood of recidivism and thereby reduce the level of harm inflicted upon the communities where ex-prisoners reside, we developed a housing mobility program for newly released prisoners called the Maryland Opportunities through Vouchers Experiment (MOVE). This randomized controlled trial (RCT) incentivizes residential relocation among recently released prisoners by providing them with a housing subsidy for 6 months of free housing.

The intent of this article is to describe the design of the MOVE program, and to provide a preliminary assessment of its effect and the feasibility of proceeding with full-scale implementation of the program. This article proceeds by first describing the evidence base upon which the MOVE program was developed. We then describe the design of the RCT and present results of a pilot evaluation.

## Existing evidence

Recent research buttresses the argument that residential change can lead to a reduction in recidivism. Kirk (2009, 2012, Forthcoming) used the neighborhood destruction in New Orleans following Hurricane Katrina as a natural experiment to investigate the effects of residential change on recidivism. He found substantial reductions in rates of reincarceration among ex-prisoners who moved away from their former parishes. Individuals who moved were 15 percentage points less likely to be reincarcerated.

Outside of the Katrina research, there is a host of quantitative and qualitative research that supports the hypothesis that residential relocation can produce a reduction in criminal behavior. For instance, in an analysis of data from the Cambridge Study of Delinquent Development, Osborn (1980) found that delinquents who subsequently moved away from London were significantly less likely to be reconvicted of a crime than delinquents who stayed in London. In a longitudinal study drawing upon survey data from the Project on Human Development in Chicago Neighborhoods, Sharkey and Sampson (2010) found that, among Chicago adolescents who moved to a different neighborhood within the city, their likelihood of violent offending increased. However, moving outside of Chicago significantly reduced violent behavior (see also Farrall et al. 2014; Keels 2008; Laub and Sampson 2003).

While there is some research evidence to support the contention that residential change can lower the risk of recidivism, it remains to be seen whether these findings, particularly those from a tragic natural experiment, can translate to a real-world policy environment. Moreover, because none of the aforementioned research relied upon a randomized experiment, it is impossible to rule out alternative explanations for the observed relationship between residential change and recidivism. To test the effects of policy interventions, a more rigorous research design is necessary. Arguably, the best method available for this purpose is an RCT. This recognition motivated the development of the MOVE program, which was designed and implemented in coordination with the Maryland Department of Public Safety & Correctional Services (DPSCS). We initiated a pilot test of the MOVE program in 2015.

## MOVE background and design

Research by the Urban Institute reveals that substantial proportions of returning prisoners would welcome the opportunity to move away from their former criminogenic environments in order to avoid further crime (see, e.g., Visher and Farrell 2005), but many do not move because of a lack of resources to move and a lack of awareness of even how to do so. The MOVE program was designed to incentivize and facilitate residential moves among exiting prisoners by providing a privately funded housing subsidy as well as housing relocation assistance to participants.

We tested two different designs of the intervention (Design 1 and Design 2) during the pilot phase of the program, as shown in Table 1. In both designs, the treatment group of movers received a housing subsidy equivalent to the U.S. Department of Housing and Urban Development (HUD) Fair Market Rent, for use only in a designated geographic area located a significant distance away from where they resided prior to incarceration. Individuals who resided in Baltimore City prior to incarceration were provided a subsidy for housing in Prince George’s (PG) County, MD, which is located adjacent to Washington, DC, approximately 40 miles from Baltimore. Individuals who resided in PG County prior to incarceration were provided a subsidy for use only in Baltimore City.

**Table 1:** MOVE research designs

	Design 1	Design 2
Treatment group	6-month subsidy to move to a new jurisdiction	6-month subsidy to move to a new jurisdiction
Control group	6-month subsidy in home jurisdiction	No subsidy
Nature of intervention (i.e., difference between treatment and control)	Moving to a new city	Moving to a new city + receipt of free housing
Sample size	6 Treatment; 9 Control	8 Treatment; 7 Control
Recruitment period	June 2015	March 2016, July 2016

Within Design 1, the control group received a housing subsidy which could be used back home in the jurisdiction where they resided immediately prior to incarceration. For instance, individuals who resided in Baltimore City prior to incarceration were provided a subsidy for housing in Baltimore. In contrast, under Design 2, the control group did not receive a housing subsidy. Accordingly, with Design 2, the control group represented the status quo with respect to the situation prisoners typically face when they are released from prison. To deliver some benefit to all individuals for participating in the program, we provided a US$100 gift card to all Design 2 participants upon release, which they could use for immediate necessities such as toiletries, clothes, and food.

### Sample size

A total of 30 participants were recruited into Design 1 and Design 2 of the program, with participants coming from four Maryland prisons, three in central Maryland and one in southern Maryland (as described in further detail below, we also compared these 30 against a non-randomized group of individuals released from prison just prior to the start of our program). We typically recruited individuals into the program roughly 3 months prior to their expected release date from prison. To be eligible to participate in the program, participants must have resided in Baltimore City or PG County prior to incarceration. The particular prisons were selected because they release a relatively large number of individuals originally from Baltimore City and PG County.

### Housing subsidies

Whereas the housing subsidies in the MOVE program were privately funded, we designed the program similar to the U.S. Department of Housing and Urban Development’s (HUD) Section 8 voucher program so that the housing subsidies would be used in the private rental market. In other words, rather than place participants temporarily in a transitional housing unit managed by the MOVE program, we facilitated residence in a private market dwelling by providing contracted housing location assistance as well as a housing subsidy. Participants signed their own lease, sometimes for a duration longer than the 6-month subsidy, and all subsidy payments were provided directly to landlords.

The MOVE program was initially designed to provide 3 months of free housing subsidies to both the mover and stayer groups. After conducting outreach to landlords, our impression was that the lack of income among our participants often presented a larger barrier to securing a lease than the stigma of a criminal record. The promise of 3 months of guaranteed rent was not sufficiently comforting to landlords looking for a 6-month or 1-year lease. Some prospective landlords worried that after the rent subsidy from the MOVE program ended, our participants would be unable to pay rent and eviction would be necessary. Accordingly, in the pilot we ultimately determined that providing a 6-month subsidy was more optimal than 3 months, and the results presented to follow are based upon an intervention with 6 months of completely subsidized housing.

The value of the housing subsidy was pegged to HUD’s established fair market rent for a one-bedroom dwelling in a given area for 2015: $985/month in Baltimore City and $1230/month in PG County. The program also provided a security deposit equivalent to 1 month’s rent. We encouraged participants not to rent an apartment for the full amount or above if they did not anticipate having enough income to pay the rent after the subsidy ended. Most did not rent at the full subsidy level, and the remaining balance was retained by the program.

Participants were not required to live alone; rather, they could choose to use the subsidy to reside with immediate family members. If a participant wished to move in with a family member with an existing lease or mortgage, we facilitated paying the rent or mortgage directly to the property owner or lender for 6 months as long as the location was within the appropriate geographic area.

A key component of the MOVE program was housing counseling and housing location assistance. To provide these services to project participants, we contracted with Quadel Consulting, a nationally recognized housing services firm with experience administering housing voucher programs for public housing authorities. Quadel worked with participants to locate appropriate and affordable housing in targeted geographic areas designated by random assignment. While the intent of the pilot was not to conduct an extended analysis of the characteristics of the neighborhood environments where participants resided, it is important to note that the barriers to housing for ex-prisoners, including discrimination by landlords, a lack of income, and high occupancy rates, meant that our participants tended to reside in lower-income areas compared to other neighborhoods in their designated jurisdiction. In short, the treatment group moved, but did not, on average, move to areas with a dramatically different socioeconomic context than if they had returned home. However, by separating an individual from a past environment, residential change may still lower the likelihood of recidivism even if the destination neighborhood is not qualitatively more advantaged and resourced than the origin neighborhood.

### Participant eligibility

For the pilot, we attempted to limit any restrictions on the sample. However, there are a few exceptions. We excluded from the sample individuals who had a detainer in another jurisdiction that would prevent their actual release from incarceration. We also agreed with the DPSCS to exclude felony sex offenders as well as individuals screened as very high risk of violent recidivism by Maryland’s Violence Prevention Initiative forecasting system. Whereas with Design 1 of the pilot, we did not restrict the age range of participants and had participants ranging in age from 22 to 53, for Design 2 we limited eligibility to individuals between ages 18 and 45 in order to target participants most at risk of recidivism (actual sample range of 19–45; see Table 2 for further descriptive information). Finally, the pilot only recruited male prisoners. Female prisoners were not eligible because of the combination of the limited number of participant slots within the small sample pilot and the relatively small proportion of the Maryland prison population accounted for by women (approximately 5%).

**Table 2:** Baseline comparison of experimental groups in the MOVE program

	Design 1		Design 2	
	Treatment	Control	Treatment	Control
% Black	100	100	100	100
Median age at release	35.5	46	29	35
Most serious commitment offense				
Violent	1.00	0.56	0.75	0.57
Property	0.00	0.11	0.00	0.00
Drug	0.00	0.22	0.25	0.43
Public order	0.00	0.11	0.00	0.00
Median prior arrests	5	12	4.5	6
Median prior convictions	3	7	2.5	4
Median prior imprisonments	3	6	2	3

The vast majority of prisoners in Maryland are released either through a decision by the parole board or through the diminution of their sentence through the accrual of good time credit. We focused our recruitment efforts on the latter group of individuals released via mandatory release because we would know with a high degree of certainty exactly when they would be released from prison, which we could then communicate to prospective landlords.

### Take-up rate and treatment compliance

In June 2015, we recruited 15 new participants into Design 1 of the program out of 17 invited to participate (88% enrollment). Six were randomly assigned to the treatment group and nine to the control group. We ultimately placed three of the six treatment group participants into housing (50%) and all nine of the control group.

As noted, we altered the design of the program in 2016 for the purposes of making a direct comparison between the treatment condition and the status quo (i.e., no housing subsidy), and also to increase the take-up rate into housing. On the latter point, the design of the program was such that we did not penalize participants for dropping out of the program. Indeed, setting some kind of penalty for dropping out would contravene the principles of ethical research. However, to avoid recruiting or enrolling people who would not actually consider moving to a new area if they were assigned to the treatment group, we redesigned the program with the intent of only recruiting participants who would legitimately consider moving to a new city. We did so by removing the subsidy for control condition participants returning to their home community (in Design 2).

We recruited six participants to the program in March 2016 under Design 2 (6 out of the 8 invited to participate enrolled, or 75%), and another nine in July 2016 (9 out of 14 enrolled, or 64% enrollment). Eight of these 15 participants were randomly assigned to the treatment group of movers and 7 were assigned to the control group. We again had a 50% take-up rate in the treatment group despite the fact that we presumably only enrolled participants who would legitimately consider moving to a new area. A detailed discussion of the take-up rate and very feasible ways to enhance it can be found in other project publications (Kirk Forthcoming).

## Pilot results

Before describing the results, it is important to bear in mind that the MOVE pilot relied upon a small sample, and that the main purpose of the pilot was to test out the procedures and design of the program so that suitable modifications could be made in advance of a full-scale implementation. Nevertheless, since a full implementation—for which we are presently seeking funding—would involve multiple years of recruitment and a 3-year follow-up period to assess recidivism, we offer descriptive pilot results as a preliminary assessment of the potential efficacy of the MOVE program.

Table 2 presents a descriptive comparison of our treatment and control groups across the two designs. A benefit of an RCT is that random assignment facilitates causal inference by making experimental groups randomly similar to each other. A sufficiently sized sample is necessary in order to produce comparable groups, which may be violated in our study. A sufficient sample size is also critical for having an adequately powered study.^2^ Comparisons in Table 2 reveal differences across groups, which is unsurprising given the small sample size. For instance, the median age in the treatment group in both designs is far younger than in the control groups (35.5 vs. 46 in Design 1 and 29 vs. 35 in Design 2). Given that recidivism tends to be substantially more likely among younger prison releasees (see, e.g., Durose et al. 2014), we would expect the likelihood of recidivism to be greater in the treatment group if there were no other differences across groups affecting recidivism.

Likely reflective of the age differences between the treatment and control groups, we also see differences in both designs in criminal history (i.e., prior arrests, convictions, and imprisonments). There are also some differences in the offense of commitment for the incarceration at the time of recruitment.

The entire sample of participants was black, although we did not restrict eligibility by race and ethnicity. The fact that our entire sample was black reflects the fact that 70% of the overall prison population in Maryland is black and that we were specifically recruiting prisoners who were originally from metropolitan areas (i.e., Baltimore and Prince George’s County) with large proportions of black population and very large proportions of black males among the convicted felon population (Pew Charitable Trusts 2015).

Recidivism rates across the two designs are presented in Table 3, with the upper set of rows of each design presenting results for the intention-to-treat analysis, which includes the non-compliers who were not placed into housing in addition to the compliers, while the lower set of rows excludes the non-compliers (i.e., the 50% of treatment group participants who did not take-up into housing). Recidivism is measured by rearrest during the first-year post-release. Rearrest data were obtained from the DPSCS, and we cross-checked these data with arrest and court records from the Maryland Judiciary Case Search online portal.

**Table 3:** One-year rearrest in the MOVE program

Design 1 (recruitment in June 2015)			
	Treatment (housing/mover)	Control 1 (housing/stayer)	Comparison (no housing)
Intention-to-treat (full sample)			
# Rearrested	0	0	5
No rearrest	6	9	18
% Rearrest	0.0%	0.0%	21.7%
Chi-square = 3.755, p value = 0.153			
Fisher's exact test = 0.241			
Z-test, Treatment vs. Comparison (one-tailed) = -1.255, p value = 0.105			
Z-test, Control 1 vs. Comparison (one-tailed) = -1.523, p value = 0.064			
Subset to those Housed (excludes non-compliers)			
# Rearrested	0	0	5
No rearrest	3	9	18
% Rearrest	0.0%	0.0%	21.7%
Chi-square = 3.044, p value = 0.218			
Fisher's exact test = 0.411			
Z-test, Treatment vs. Comparison (one-tailed) = -0.899, p value = 0.184			
Z-test, Control 1 vs. Comparison (one-tailed) = -1.523, p value = 0.064			
Design 2 (recruitment in March and July 2016)			
	Treatment (housing/mover)	Control 2 (no housing)	
Intention-to-treat (full sample)			
# Rearrested	2	4	
No rearrest	6	3	
% Rearrest	25.0%	57.1%	
Chi-square = 1.607, p value = 0.205			
Fisher's exact test = 0.315			
Z-test (one-tailed) = -1.268, p value = 0.102			
Subset to those Housed (excludes non-compliers)			
# Rearrested	1	4	
No rearrest	3	3	
% Rearrest	25.0%	57.1%	
Chi-square = 1.061, p value = 0.303			
Fisher's exact test = 0.545			
Z-test (one-tailed) = -1.030, p value = 0.152			

In Design 1, participants were only randomly assigned to the Treatment or Control groups. The Comparison group represents eligible participants who were released from prison just prior to the start of recruitment. Including this group allows us to compare the Treatment and Control groups against the status quo (i.e., no housing subsidy)

In Design 1, we find that none of the treatment group of movers were rearrested within the 1-year follow-up period, whether in the full sample or the subset of compliers. Similarly, none of the control group of stayers were rearrested.

In order to compare these percentages from the treatment and control groups against the status quo (i.e., exiting prisoners who do not receive a free housing subsidy), we made use of a non-random comparison group of 23 individuals who did not receive a housing subsidy. The column in Table 3 under Design 1 labeled “Comparison” consists of a group of prisoners that we deemed eligible to participate in the program but ultimately did not attempt to recruit. This occurred because 23 individuals we deemed eligible for recruitment in 2015 were released by the time we finalized our planning and managed to get the first recruitment visit scheduled with prison staff. These individuals were never told about the program by the project team or prison staff, but we would have attempted to recruit them if we had scheduled the prison visit sooner. Given our high rate of enrollment (77%), we would expect that we would have enrolled a vast majority of these 23 individuals. Nearly 22% of this non-random comparison group were rearrested. The differences across groups suggest that the receipt of free housing, or the combination of free housing and residence in a new city, yields a reduction in the likelihood of recidivism.

For the sake of thoroughness despite the lack of power in our analysis, we conducted hypothesis testing across the three groups via Chi-square tests as well as Fisher’s exact test, the latter being appropriate for small samples such as ours. Unsurprisingly given the small sample size, we do not find evidence of a statistically significant association in either test. To test directional hypotheses that both residential change and receipt of housing assistance lower the likelihood of rearrest, we also conducted two-group comparisons using one-tailed Z-tests. Even with a small sample size, these tests reveal some evidence of marginally significant differences between the comparison group and the treatment and control groups respectively (p-values of 0.105 and 0.064 for the full sample).

In Design 2, the treatment group had a recidivism rate of 25% in both the intention-to-treat analysis and the analysis subset to the compliers. In fact, for the treatment group under both Designs 1 and 2, the percentage rearrested is the same for the intent-to-treat effect as for the treatment-on-treated effect. Whether this consistency holds in a full implementation is a finding we will closely monitor once the research gets to that stage, as it has important implications for understanding the efficacy of the treatment and the mechanisms underlying any effect.

By comparison, the control group in Design 2 had a recidivism rate of 57.1% (four out of seven).^3^ Even with such a large difference between the treatment and control groups (.321), our sample size of 15 participants yields an estimated statistical power of just 0.234. Chi-square and Fisher’s exact tests do not provide evidence of a statistically significant treatment effect, but the lack of power certainly suggests that our tests may be affected by Type II error. Put simply, our small sample size and lack of power likely mean that we have retained our null hypothesis (no effect) when it should be rejected. A one-tailed Z-test that rearrest is lower in the treatment group than the control group equals -1.268 in the full sample analysis, with a p-value just above the cutoff of 0.10 typically used to denote marginally signficant relationships.^4^

## Discussion

Upon exiting prison, formerly incarcerated individuals typically return to the same general areas where they resided before incarceration. The reasons ex-prisoners return to home jurisdictions are many, and include family and social ties as well as familiarity and attachment to particular places. However, barriers to securing housing, such as a lack of income, discrimination in the private housing market, the denial of public housing and vouchers to certain classes of felons, and the lack of affordable housing in the U.S. more generally, mean that many former prisoners will return to their home neighborhoods even when they do not want to because they simply have nowhere else to go.

MOVE is a housing mobility program for formerly incarcerated individuals designed to examine the effect on criminal recidivism of residential relocation away from former neighborhoods and cities. The intent of this article has been to describe the design of the program, and to provide a preliminary assessment of its feasibility and efficacy. With the important caveat that these findings are based on a small sample, the descriptive results presented in Table 3 preliminarily suggest that there may be some benefit to moving and also a benefit to obtaining free housing irrespective of the location. Recidivism was descriptively lower among the treatment group than the control group in Design 2, and was lower for non-movers who received free housing versus non-movers who did not receive housing (the comparison between Control 1 and the Comparison group in Table 3).

A clear and obvious limitation of this study is the small sample. The MOVE pilot revealed that the challenges of implementing the program are not insurmountable and could be remedied through slight modifications to the program design. The next step in this effort is to proceed with full-scale implementation and evaluation, including a cost–benefit analysis and a thorough assessment of neighborhood conditions across the experimental groups.

Ultimately, if the hypotheses underlying the program are supported, then policies that provide greater access to housing assistance for formerly incarcerated individuals may be worth pursuing. Whereas the combination of stable housing and residential change may be particularly advantageous, mounting research evidence suggests that stable housing on its own is important for reducing the risk of recidivism (see, e.g., Steiner et al. 2015). One idea is to provide housing programs and opportunities directly to people with criminal records (see, e.g., Hamilton et al. 2015). One method to fund such programs, in the spirit of justice reinvestment, is to reinvest savings from reduced rearrest and reincarceration into housing subsidies for formerly incarcerated individuals. An alternative idea is to expand affordable housing opportunities for low-income households in general, some of which will include formerly incarcerated individuals.

The National Low Income Housing Coalition offers one possible strategy for expanding affordable housing development.^5^ Reducing the upper limit on the proportion of a homeowner’s mortgage eligible for interest deduction on federal taxes from $1 million to $500,000 would yield additional tax revenue which could then be used to fund the national Housing Trust Fund for building affordable homes as well as rental assistance programs for low-income groups. The counter argument to this proposal is that the mortgage interest deduction is critical for spurring homeownership, yet recent research convincingly reveals that such an argument lacks empirical support (Gruber et al. 2017). Whereas the current U.S. presidential administration has indeed proposed to reduce the mortgage interest deduction as part of a pending tax reform package, the savings would be directed to a tax cut largely benefitting the wealthy and corporations rather than put towards affordable housing development for lower-income groups. Whatever the method used to expand low-income housing opportunities, results from the present study preliminarily suggest that providing stable housing for the formerly incarcerated, including if the location of the housing allows for a fresh start in life, will help reduce the harm from the stubbornly high rates of recidivism that continue to plague U.S. communities.

## Footnotes

1 The lack of sufficient separation between old and new residences may explain the disappointing findings from the Moving to Opportunity (MTO) housing mobility demonstration with respect to problem behavior. For instance, the long-term impact evaluation revealed no statistically significant difference in arrests for the experimental group relative to the control group (Sanbonmatsu et al. 2011). It may be the case that the residential moves in the MTO program were not, on average, substantial enough to produce a turning point in behavioral trajectories. Indeed, Sampson (2008) found in his analysis of the Chicago MTO site that a vast majority of MTO experimental families moved a relatively short distance from their origin neighborhoods.

2 Using Kirk’s (2009, 2012) quasi-experimental results from Louisiana as a guide, which revealed a 15 percentage point difference in recidivism between movers and non-movers, statistical power calculations indicate that a sample size of 30 can detect a 15 percentage point difference in recidivism in favor of the treatment group of movers with just 21% statistical power (one-sided). Of course, whereas the treatment in Kirk’s studies was residential change, the treatment in the second design of the present RCT is a combination of residential change and receipt of a housing subsidy, which may lower the likelihood of recidivism even further.

3 The differences in rearrest rates between Designs 1 and 2 of the RCT likely reflect, in part, the differences in the age distribution of the samples.

4 At the suggestion of the editors, we also conducted permutation tests with the Chi-square test and Z-test. Accordingly, we reshuffled the treatment labels in our data by reassigning an observation to one of the other observed treatment values in the data (i.e., for each shuffle, a treatment observation would have his group assignment randomly reshuffled to be that of one of the other seven treatment group participants, or one of the seven control group participants). Our inferences from these permutation tests are consistent with inferences from our Chi-square tests and Z-tests, and we would again note that all analyses are underpowered.

5
http://www.unitedforhomes.org/
